# The Role of the Tumor Microenvironment in Gastroenteropancreatic Neuroendocrine Tumors

**DOI:** 10.3390/ijms26125635

**Published:** 2025-06-12

**Authors:** Srujana V. Yellapragada, Steven D. Forsythe, James P. Madigan, Samira M. Sadowski

**Affiliations:** 1 Neuroendocrine Cancer Therapy Section, Center for Cancer Research, National Cancer Institute, National Institutes of Health, Bethesda, MD 20892, USA; srujana.yellapragada@nih.gov (S.V.Y.); steven.forsythe@nih.gov (S.D.F.); james.madigan@nih.gov (J.P.M.); 2 Surgical Oncology Program, Center for Cancer Research, National Cancer Institute, National Institutes of Health, Bethesda, MD 20892, USA

**Keywords:** Gastroenteropancreatic neuroendocrine tumors (GEP-NETs), Tumor Microenvironment (TME), biomarkers, treatments

## Abstract

Gastroenteropancreatic neuroendocrine tumors (GEP-NETs) are a family of tumors that arise throughout the gastrointestinal tract. These tumors are heterogeneous, with complex clinical symptoms and tumor behaviors, and demonstrate rising incidence rates worldwide. In addition to their nature, GEP-NETs possess limited diagnostic and therapeutic options, which results in poor survival rates for patients with metastatic tumors. Given these findings, a further analysis of these tumors’ biology is needed to determine new therapeutic strategies. The tumor microenvironment (TME) consists of several residual cell populations and non-cellular components whose altered behavior creates a tumor-supportive niche. Studies from other cancers demonstrate the TME’s significance in tumor initiation, progression, and spread. In this review, we discuss efforts to characterize the TME in GEP-NETs. Preliminary studies of the immune system in GEP-NETs have led to several major clinical trials, with limited success. Efforts to target signaling crosstalk between cancer-associated fibroblasts, vascular endothelial cells, and tumor cells has led to major discoveries and multiple approved therapies. Finally, alterations to the extracellular matrix may lead towards an improved understanding of GEP-NET development, behavior, and improved detection methods. While research has rapidly expanded our knowledge within the last decade, further work is needed to bring our understanding of the GEP-NET TME in line with other rare cancers.

## 1. Introduction

Neuroendocrine tumors (NETs) are neoplasms initiating from neuroendocrine cells throughout the body. They represent 2% of all malignancies in the United States [1]. While some are of unknown origin, the most prevalent sites arise from those of the gastrointestinal tract. These tumors, arising in the stomach, bowel, and pancreas, belong to a subgroup known as gastroenteropancreatic neuroendocrine tumors (GEP-NETs) [2]. Regardless of origin, GEP-NETs are classified based on tumor differentiation (the resemblance of the tumor tissue to its normal tissue counterpart) and tumor grade (associated with the rate of tumor cell replication). Based on tumor differentiation, GEP-NETs are divided into three categories: well-differentiated, poorly differentiated, and mixed. Well-differentiated GEP-NETs contain a uniform population of tumor cells with round nuclei and “salt and pepper” like chromatin. Poorly differentiated tumors possess dispersed chromatin, nuclear molding/smudging, and scant cytoplasm. Tumor grade is determined by the mitotic rate and/or the Ki-67 proliferation index of tumor cells. Tumors with a mitotic rate of <2, with a Ki-67 proliferation index < 3%, are considered low/grade 1 tumors. Mitotic rates between 2 and 20 and a Ki-67 index of 3–20% are intermediate/grade 2 tumors, while mitotic rates of >20 and Ki-67 of >20% are high/grade 3 tumors [3]. Additionally, GEP-NETs are genetically heterogeneous. Well-differentiated NETs often have a low tumor mutational burden, with observable mutations in MEN1, DAXX, and ATRX [4]. NETs are also classified based on hormonal secretions. Those that secrete hormones are labeled as “functional”, where patients are often diagnosed with an NET at earlier stages, given the symptoms that arise from the hormones. Tumors that do not secrete hormones are considered “non-functional”. Patients with these silent tumors are often diagnosed at later stages, as symptoms do not arise until metastasis formation.

Gastric NETs (GasNETs) represent 5–23% of all digestive neuroendocrine neoplasms and have an incidence rate of 6.98 per 100,000 cases and an 86.7% five-year survival [5]. Based on the characteristics discussed in the previous section, GasNETs are classified into four types. Type 1 and 2 GasNETs are gastrin-secreting tumors, known as gastrinomas, while types 3 and 4 are sporadic, with a higher related mortality [6]. Small intestinal neuroendocrine tumors (SiNETs) make up 1–2% of all gastrointestinal malignancies. They have an annual incidence rate of 1 per 100,000 cases and arise from various locations of the small bowel, including the jejunum, ileum, and duodenum. Duodenal NETs can be sporadic but are often found among patients with ZES and MEN1 syndromes. They have a 5-year and 10-year survival rate of close to 85% and 95%, respectively, but only 10% for those with metastases. Most SiNETs arising from the jejunum and ileum are nonfunctioning, with 20% of patients presenting with liver metastases. Overall, their 5-year survival rate is 65%, but this is lower at 36% for patients with metastatic tumors. SiNETs are highly genetically heterogeneous, with mutations in VHL, BRAF, FGFR2, MEN1, MLF1, SRC, SMAD, and FANCD2 genes [7].

Pancreatic neuroendocrine tumors (PNETs) are the second most common malignancy in the pancreas and have a rising incidence, with 3000 new cases diagnosed in the United States every year. Patients with functional PNETs are diagnosed at earlier stages, given the symptoms that arise from hormone production. However, most PNETs are considered non-functional. The five-year overall survival rate for localized PNETs is 83%, while that of metastatic PNETs is 28% [8].

Due to their individual characteristics, GEP-NETs are a group of heterogeneous diseases, with variable treatment options depending on the site of origin, aggressiveness, and functional status, with surgery being the primary treatment [9]. As a result, the effective treatment of GEP-NETs requires in-depth knowledge of both tumor and non-tumor factors, which contribute to their progression and may serve as therapeutic targets. While there have been considerable recent efforts to better model GEP-NETs, there is also a need to study the non-tumor cell components of GEP-NETs [10,11]. The tumor microenvironment (TME) is complex, as it houses not only tumor cells but also residual non-tumor cell populations, which promote tumor development. These cell populations comprise immune cells (both innate and adaptive), cancer-associated fibroblasts (CAFs), and vascular cells. These TME cell types interact with each other through the secretion of factors that regulate specific cellular pathways or alter the gene expression of the cells and are often referred to as secreted biomarkers. Lastly, the TME is composed of the extracellular matrix (ECM), which houses TME cells and interacts with them to promote the TME’s structural and mechanical properties for tumor progression. Understanding the interplay of the TME with the tumor will allow for a more complete image of GEP-NET development and may help explain the complexity of these tumors.

Characterizing the TME is vital for determining the efficacy or resistance to certain anti-tumor treatments and may lead to the discovery of alternative and effective therapeutic targets for GEP-NETs. Studies among other gastrointestinal cancers, such as colorectal cancer and pancreatic adenocarcinoma, have showcased how the TME influences tumor progression and treatment response [12]. As with these prior studies, investigating the TME of GEP-NETs would assist in the enhancement of their therapeutic treatment.

In this review, we detail the components of the TME in GEP-NETs, how their dysfunction relates to clinical outcomes, and highlight the efforts to investigate these components in the research setting and efforts to target them.

## 2. Components of the Tumor Microenvironment

### 2.1. Immune Cells

**Figure 1:**
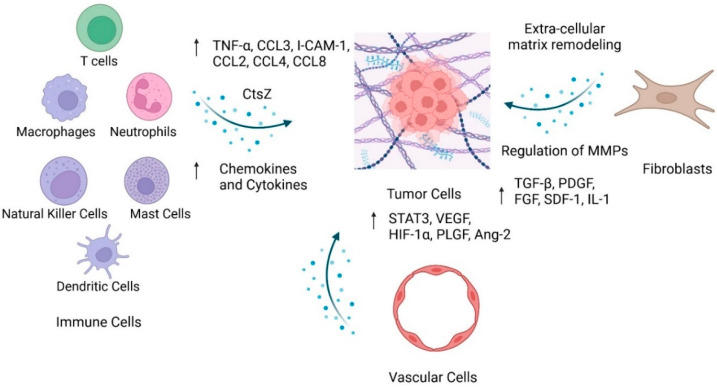
Residual cell population interacts with tumor cells within the tumor microenvironment. Image created with Biorender.com.

Immune cells are derived from hematopoietic stem cells, located in the bone marrow, and travel through the body’s blood and tissue to fight against pathogens. Innate immunity is the first line of defense in the immune response by attacking common foreign invaders and preventing local infections from becoming systemic [13]. However, more specialized targeting is required for uncommon or difficult-to-detect foreign bodies, including some cancer cells. As a result, the adaptive immune response is initiated for additional specific targeting. This adaptive response, however, is not activated immediately. The tumor immune microenvironment (TIME) is composed of innate immune cells: macrophages, neutrophils, mast cells, myeloid-derived suppressor cells, dendritic cells, natural killer cells, and the adaptive immune cells, T and B lymphocytes (Figure 1). In cancer, these immune cells play a dual role in promoting and preventing tumor and metastasis development [14]. It is crucial for the immune response to prevent tumor cells from spreading, leading to advanced disease. To date, the TIME of GEP-NETs is understudied, delaying new discoveries to exploit immune cell populations for targeted treatments.

#### 2.1.1. Macrophages

Macrophages are an important component of the body’s immune system defense against pathogens through maintaining proper tissue homeostasis and remodeling. Their other immune defense roles include phagocytosis, antigen presentation, and T-cell activation by producing chemokines and cytokines. In response to the nature of the pathogens, macrophages can undergo polarization into either the “classically activated” M1 subtype or the “alternatively activated” M2 subtype [15]. M1 macrophages have enhanced phagocytic activity, antigen-presenting capacity, and an increased production of proinflammatory cytokines. M2 macrophages promote angiogenesis and neovascularization, stromal remodeling, and tissue repair. In the context of cancer, M2 macrophages secrete factors that facilitate cancer cell immunosuppression, proliferation, invasiveness, epithelial–mesenchymal transition, and metastasis formation, negatively affecting patient prognosis [16]. Thus, macrophages in the TME are considered key modulators in cancer progression and are often referred to as tumor-associated macrophages (TAMs).

In PNETS, TAMs have been associated with an increased Ki-67 index, tumor recurrence, and the presence of metastatic lesions [17,18]. Higher TAM density has also been linked with increased PD-L1 expression in high-grade GEP NETs; PD-L1 surface-levels on tumor cells are often associated with poor prognosis and immunotherapy resistance [19]. Understanding TAM involvement in NET development may lead to a better understanding of tumor behavior and improved clinical perspective. Some studies present TAMs’ interactions with blood stimulation proteins, including colony stimulating factor 1 (CSF-1), for the development of this population in the TME. Studies demonstrate a deficiency in this protein, leading to a decrease in the number of pro-tumorigenic macrophages. Among pre-clinical studies in GEP-NETs, mice deficient in CSF-1 were shown to develop fewer PNETs compared to wild-type mice [20]. Some studies present TAM secreting factors associated with tumor progression, including cathepsin Z (CtsZ), a protease which promotes tumor proliferation and invasion [21]. Researchers were able to demonstrate that CtsZ activity is mediated by integrin interactions and the ECM through the Arg-Gly-Asp (RGD) motif in mouse models. Other studies aim to elucidate the process of macrophage polarization in NETs, particularly towards the M2 phenotype. So far, it is observed that exosomes, membrane-bound vesicles and messengers secreted by tumor cells, may play a role in this process. This observation was made among hypoxic PNET cell-derived exosomes, such as CEACAM5, in PNET progression, making it a potential diagnostic marker (Table 1) [22].

**Table 1:** Summary of secreted factors by cell population in TME for GEP-NETs.

Cell Population	Tumor-Supportive	Tumor-Suppressive
Tumor-Associated Macrophages (TAMs)	Colony stimulating factor (CSF-1) [20] Cathepsin Z (CtsZ) [21] CEACAM5 [21] Tie-2 [23]	
Tumor-Associated Neutrophils	Cyp46a1 [24] Hypoxia inducible factor 1-α (HIF 1-α) [24]	
Mast Cells	Stem cell factor (SCF) [25] Myc [26] Bruton tyrosine kinase (Btk) [26]	
Dendritic Cells	Toll-like receptor 3 (TLR3) [27] FMS-like tyrosine kinase 3 ligand (FLT3LG) [28]	
T Lymphocytes	PD-1/PD-L1 pathway [24] HHLA2 [29] H4 (B7x) [29]	CD47 [30]
Vascular Endothelial Cells (VECs)	Heparanase [31] VEGF/VEGFR-2 [32] CYR61 [32] VEGFC [33] VEGFR-2 [33] VEGF-B [34] RSUME [35] Cut homeobox 1 (CUX 1) [36] PDGFRα and PDGFRβ [37,38]	Neutropilin-2 (NRP-2) [34]
Cancer-Associated Fibroblasts (CAFs)	mTOR [39] STAT3 [40] TGF-β (β-1, β-2, β-3) [41] PDGF [42] bFGF [43,44] Thrombin, bombesin, bradykinin, and vasopressin [45] 5-HT’s [46] Stromal cell-derived factor-1 (SDF-1) [47]	

Though macrophages are shown to be a prognostic biomarker for PNETs, they are also capable of providing therapeutic targets for treatment. For example, Tie-2 is a tyrosine kinase receptor that is found to be expressed on macrophages in mouse models of metastatic PNETs. Discovering the expression of Tie-2 allows researchers to utilize a kinase switch control inhibitor, Rebastinib, to inhibit Tie-2 and effectively reduce tumor volume and metastasis (Table 1) [23]. Macrophage phenotypic variances likely have a great influence on the efficacy of specific therapies. This observation was demonstrated by a differential gene expression analysis of 600 immune-related genes across a cohort with varying molecular subtypes: a metastasis-like primary (MLP)-1 and second (MLP)-2 where (MLP)-1 is insulinoma-like [27]. They identified higher levels of immune-related genes, including those associated with M1 macrophage-mediated immune escape mechanisms, in MLP-1 compared to MLP-2. Another study assessed the efficacy of liposomal clodronate treatment of TAMs, using in vitro and RIP1Tag2 mouse models, and found reduced TAM infiltration and micro vessel density. These results suggest that liposomal clodronate treatment could be a novel PNET therapeutic approach and requires further validation [48].

The subtypes of macrophages are well-established and observed in GEP-NETs. There are also pre-clinical studies that identify protein targets that may be associated with TAM infiltration, proliferation, and tumor progression. However, more studies need to be performed to validate the efficacy of these targets in translational work. Similarly, promoting reverse macrophage polarization is another strategy considered towards the reduction in TAM infiltration; however, further studies are needed to demonstrate specific factors or pathways that promote this mechanism in GEP-NETs. So far, macrophage studies are primarily focused on PNETs. A few therapeutic treatments, including rebastinib and liposomal clodronate treatment, show potential for preventing TAM activity with PNET progression, but further pre-clinical studies are also needed for the other GEP-NET subtypes.

#### 2.1.2. Neutrophils

Neutrophils constitute over half of the myeloid-derived white blood cell population. They attack pathogens during the initial innate immune response; however, they are also capable of causing tissue damage during this process. Tumor-associated neutrophils (TANs), neutrophils within solid tumors, are classified into two phenotypes: anti-tumor (N1) and pro-tumor (N2) [49]. N1s are characterized by elevated levels of TNF-α, CCL3, and ICAM-1, while N2s are often characterized by an upregulation of CCL2, CCL4, and CCL8. The high intra-tumor density of N2 neutrophils in the TME correlates with lymph node metastasis and an increase in tumor grading. While N1 cells aim to suppress tumor growth and metastasis through cytotoxic activity and the activation of immunosuppressive cells, N2 cells may promote tumor growth and angiogenesis through the secretion of ECM remodeling enzymes and pro-angiogenic factors. Studies have shown that PNET and GasNET patients possess a higher neutrophil–lymphocyte ratio (NLR) and neutrophil counts compared to normal volunteers [18,50]. Similarly, tumorigenesis among SiNETs was correlated with the accumulation of N2-like neutrophils [51]. This observation was in correlation with increasing tumor stage, grade, and the presence of lymph nodes or perineural invasions. While this phenomenon is not fully understood in SiNETs, these findings may be due to increased levels of circulating lipopolysaccharide, which promotes the upregulation of the C3a receptor on accumulated neutrophils. Among PNETs, TANs assist with neoangiogenesis and tumor growth through the simultaneous overexpression of the enzyme Cyp46a1 and regulation of hypoxia inducible factor 1-α (HIF 1-α) (Table 1). These actions led to the generation of tumor-derived oxysterols, which recruit neutrophils for pro-tumor activity [52]. NLR is also an independent prognostic factor for worse recurrence-free and overall survival [50]. Additionally, neutrophil and macrophage extracellular traps, mesh structures composed of a DNA skeleton and proteins of immune cells that remain after their cell death process, can also be biological indicators of patient prognosis, as shown among non-functional PNETs [53].

Further research is needed to translate these observational studies into potential therapeutic opportunities. It is established that GEP-NET patients possess a higher neutrophil count compared to normal individuals, and there are markers identified to determine the presence of both neutrophil subtypes. However, further work is needed to answer whether high TAN count relates to GEP-NET growth. This may require an analysis of receptor expression on neutrophils that interact with the tumor cells, their associated pathways, or secreted factors of neutrophils to pursue this question. While this was observed among SiNETs and PNETs through the observations of C3a upregulation and Cyp46a1 overexpression, respectively, there needs to be a validation of these targets to conclude their relationship with tumor growth. Lastly, the presence of neutrophil and macrophage extracellular traps among PNETs raises curiosity about the crosstalk of TAMs with TANs. There should also be studies to observe the TAN population in response to TAM targeting and vice versa and how it correlates to GEP-NET progression.

#### 2.1.3. Natural Killer Cells

Natural Killer (NK) cells are innate lymphoid cells that contribute to immune responses against pathogens through their cytolytic activity [54]. They can kill cells directly and often target cells undergoing infection or cancer cells. Recent discoveries about healthy NK cell hindrance include the activity of surface receptors, such as killer immunoglobulin-like receptors (KIRs) and T cell immunoreceptor, which can be hijacked by cancer cells to alter NK cell biology and decrease their activity. This altered NK biology and activity can cause the NK cells to promote tumor progression instead. Among NETs, NK anti-tumor response is generally observed to be diminished. A study performed among GEP-NET patients aiming to increase NK cell cytotoxic activity, using IFN-gamma, discovered that patients with functional non-gastrinoma tumors presented higher anti-tumor response [55]. However, regardless of the variance in NK cell activity, the changing rates do not correlate with liver metastasis. Another method of increasing NK cell anti-tumor activity was demonstrated in PNETs using the oncolytic vaccinia virus, mpJX-594, with anti-programmed death receptor-1 antibody (aPD1) (mpJX + aPD1) [56]. In addition to recruiting NK cells to the TME, this therapeutic approach was able to convert a “cold” TME, a low immune-infiltrated microenvironment, into a “hot” TME, a high immune cell-infiltrated environment, with the characteristics of suppressing cancer cell proliferation and the necrosis of tumor cells.

There are few studies on the role of NK cells in GEP-NETs. However, studies performed among other cancer types regarding the role of KIRs or other receptors in supporting NK cell pro-tumor activity can be an initial analysis for these rare tumors. This observation can help lead to alternative targets of interest and mechanisms. While strategies of increasing NK anti-tumor roles are observed in PNETs through the (aPD1) (mpJX + aPD1) approach, the efficacy of this treatment should also be studied among SiNETs and GasNETs, which can be determined through the same analysis of “cold” to “hot” TME transition. If effective, further validation should be considered among pre-clinical models. While this treatment is proven to target NK cells, it is still unknown if there is a relation with other pro-tumor immune cell activity, including TAMs. Another question that arises is the variation in immune cell subtypes between primary tumors and metastatic tumors. This question refers to an observation made among NK cell populations in response to IFN gamma between primary and liver metastasis. Importantly, further studies should also consider other immune cell populations of interest.

#### 2.1.4. Mast Cells

Mast cells are basophil granule-like cells derived from the myeloid lineage. Mast cells are often the first line of defense in detecting foreign bodies and releasing inflammatory cytokines, in addition to recruiting other cells, such as NK cells, through differential cytokine release. Like other immunosuppressive cells, mast cells have both anti- and pro-tumor functions. In a pro-tumor function, when recruited to the TME through chemoattractant factors, such as stem cell factor (SCF), mast cells release several cytokines and growth factors used to promote tumor cell proliferation and angiogenesis [57]. Mast cell recruitment, promoting tumor cell expansion, often occurs through the overexpression of Myc, a proto-oncogene that regulates cell growth and cell cycle progression. While there are no clinically approved drugs for blocking mast cell degranulation, studies have shown that inhibiting Bruton tyrosine kinase (Btk) may reduce this action among pancreatic insulinomas, leading to tumor regression [58]. This is performed by inhibiting tumor stroma remodeling through the Myc, expansion of the pancreatic β cell compartment, and proliferation of the endothelial cell compartment (Table 1).

Future studies should aim for a better understanding of the role of mast cells among GEP-NETs. There are limited studies within this cell population. In particular, future analysis should consider the role of SCF, other chemoattractants, or secreted factors established in other cancer types as the initial strategy among GEP-NETs. While identifying mechanisms associated with mast cell recruitment is another strategy of interest, there is very little identified besides Btk-dependent Myc expression. Furthermore, this mechanism is only studied among PNETs and should be considered in other subtypes.

#### 2.1.5. Myeloid-Derived Suppressor Cells

Monocytic myeloid-derived suppressor cells ((M)-MDSCs) are immature myeloid cells derived from bone marrow hematopoietic precursor cells. This subgroup of cells promotes both immunosuppressive and pro-tumor activity through several functions [59]. (M)-MDSCs prevent T cell and NK cell anti-tumor activity through the production of nitric oxide (NO) and upregulation of programmed death-ligand 1 (PDL-1). Furthermore, production of cytokines and growth factors by (M)-MDSCs inhibits other immune cell responses, favoring anti-tumor activity. Patients with GEP-NETs possess high levels of circulating (M)-MDSCs [60]. In addition, patients with metastatic lesions express a higher frequency of (M)-MDSCs, in both peripheral blood and primary NEN tissues, marking a correlation between (M)-MDSC levels and tumor metastasis and showing potential utility as a biomarker. Given the relationship of (M)-MDSC with T cells, (M)-MDSCs may help determine T cell infiltration among high-grade NENs. In low-grade tumors, the CD209/DC-SIGN phenotype may identify cells with T cell suppressive activity [61,62]. These patients demonstrate limited T cell activity, showcasing the activation of the adaptive immune system with the increased infiltration of MDSCs, among NEN G3 tumors.

Despite these clinical findings, additional studies are needed to better understand the relationship between MDSCs and T cell activity, as well as methods to improve their activation. Furthermore, an analysis of (M)-MDSC interactions with other immune cells, including NK cell activity, is necessary, with the current literature having already established a relationship. Though patients with primary GEP-NETs or metastatic lesions possess a high frequency of (M)-MDSCs, there needs to be studies demonstrating the effect of this cell population on tumor growth, followed by studies on the validation of possible therapeutic targets belonging to (M)-MDSCs, as it is still unknown.

#### 2.1.6. Dendritic Cells

Dendritic cells (DCs) are a group of antigen-presenting cells (APCs) that present self and non-self-antigens for T cell priming. This function is important in the context of cancer, as it promotes T cell response for the elimination of pathogens. To sense these antigens, DC subtypes utilize recognition receptors that spot damage associated with malignant cells. After recognition and activation, DCs transport antigens to the tumor-associated draining lymph node for T-cell activation [63]. Among high-grade GEP-NETs (grades 2 and 3), classical dendritic cells (cDCs) and T cells are abundant. Increased FMS-like tyrosine kinase 3 ligand (FLT3LG) mRNA expression has been shown to detect both populations [64]. As a result, FLT3LG mRNA can be used as a biomarker for quantifying the infiltrating populations and delineating higher-grade GEP-NETs (Table 1). Other studies characterizing the immune populations of the metastatic TME have shown that there is a reduced infiltration of immature DCs, while additional immune populations are upregulated [65]. In contrast, studies have shown the increased expression of toll-like receptor 3 (TLR3) among DCs, indicating their abundant penetration (Table 1) [27]. Therefore, more information is needed regarding the DC populations between primary and metastatic GEP-NETs. In addition to their use as a biomarker, current research also offers novel DC-based immunotherapies, including the expansion of primed DCs and the creation of tumor-associated DC vaccines to activate dendritic cells for anti-tumor response [66]. Preclinical work has demonstrated the tumor-specific targeting of NET cell lines, including QGP-1, using primed DCs [67]. There have even been previous case reports of autologous DC infusion to treat NETs [68]. However, further work is needed to improve T cell responses influenced by DC activity and ensure the specific targeting of NETs. This includes a further analysis of the T cell and DC crosstalk. Studies should also consider the crosstalk of other immune cell populations, especially between primary GEP-NETs and their metastatic lesions. Regarding the present therapeutic strategies involving DCs, primed DCs appear to be a promising DC-based immunotherapy. However, further validation is needed among pre-clinical models of GEP-NETs to ensure accurate targeting strategies. An example would include targeting TLR3 and its effect on tumor progression, given that studies have established its expression on DCs.

#### 2.1.7. T Lymphocytes

T lymphocytes (T cells) are key members of the adaptive immune system and promote anti-tumor response by attacking tumor cells. T cells begin in their inactive form, referred to as naïve T lymphocytes, and contain T cell receptors (TCRs) that are responsible for antigen recognition and T cell activation. After activation, T cells can be classified by the expression of either CD4 or CD8 proteins. CD4 proteins assist in the interaction of antigen-presenting cells with the T cells for activation only among major histocompatibility complex (MHC) II molecules. Once CD4+ T cells are activated, they work to recruit other immune cells to the site of tumor cells and may directly eliminate the tumor cells through the production of various cytokines. CD8+ T cells can recognize specific exogenous antigens that CD4+ cells cannot, such as those from MHC I. CD8+ T cells work directly to target tumor cells by recognizing MHC I receptors on the tumor cell surface through the activation of the TCR complex. After the further activation and immunorecognition of tumor surface markers, CD8+ T cells release several cytotoxic granules, including granzymes and perforins, to kill tumor cells [69].

Given their role in anti-tumor activity, significant focus has been placed on clinical efforts to understand and utilize T-cells in treating cancer. Patients with distant metastatic NETs possess elevated levels of tumor-infiltrating T-cells [70,71] While elevated levels of intratumor T cells have been observed, it is known that tumors are capable of downregulating T cell activation through immune checkpoint evasion. As a result, immune checkpoint inhibitors have been considered for GEP-NET therapy. For example, PD-1 is an immune checkpoint molecule expressed on T cells, and studies have suggested inhibition of PD-1/PD-L1-pathway among higher-grade PNETs as an effective tool to improve function among cytotoxic T cells [24]. Another promising checkpoint is the B7 family, such as HERV-H LTR-Associating Protein 2 (HHLA2) and H4 (B7x). These proteins correlate with higher grades and metastasis among GasNETs and PNETs (Table 1) [29]. Experiments in cell lines and mice to reduce B7x expression demonstrated slower cell growth, decreased tumor formation, and increased T cell tumor infiltration. In addition to immune checkpoints, other cell surface targets have been studied. CD47 is highly expressed among cancer stem cells, including PNETs, and often interacts with SIRPα found on macrophages. When CD47 is expressed on the tumor cells, T cell mobilization is blocked. However, studies have shown that therapeutic applications can be enhanced when CD47 is blocked with monoclonal antibodies. For PNETs, blocking the CD47-SIRPα interaction promotes macrophages to engulf tumor cells in addition to priming for T cell activity. Though this antibody shows potential for treating PNETs, it is also observed that CD47 is co-expressed with the proto-oncogene MET, enriched by CD90^hi^ cells (Table 1). Future research may consider this cell expression for greater treatment efficacy against PNETs [30].

SiNETs and their lymph node metastasis share similar inflammatory cell profiles, where CD3+ and CD8+ T cells were particularly low in abundance. While there are limited studies on these therapeutic strategies for SiNETs and their biology in general, it is observed that metastatic lesions present an expansion of tumor-infiltrating lymphocytes [72,73]. Furthermore, exposure to autologous tumor cells leads to increased T lymphocyte degranulation. Despite these findings, the landscape of T cell therapy for SiNETs is still largely unknown. Many T-cell-based immunotherapy studies show promising results for the treatment of PNETs; however, more needs to be carried out among other GEP-NET types. Some studies demonstrate a correlation between immune checkpoint expression and increasing numbers of metastatic lesions. Therefore, studies comparing the immune checkpoint profiles and T cell subtypes between primary and metastatic tumors should be considered.

#### 2.1.8. B Lymphocytes

In contrast to the numerous studies focused on T lymphocytes among GEP-NETs, limited studies have focused on B lymphocytes. B lymphocytes are integral to the adaptive immune response in their role as the producers of antibodies following exposure to pathogens. This allows for the recognition and rapid response to previous infections, paving the way for numerous medical technologies which seek to mimic this biological function. It has been observed that CD20+ B cells are rare in PNETs and SiNETs [25]. Furthermore, the presence of CD20+ B cells in tertiary lymphoid structures has been identified as a positive prognostic factor in lower-grade non-functional PNETs [26]. Additionally, lower peripheral B cell counts were observed in PNETs, and this indicated worse progression-free survival [70]. Among SiNETs samples, single-cell RNA sequencing identified two different B cell subpopulations, distinguished by differentially expressed cycle genes. Those genes include MKI67, TOP2A, CDK1, along with canonical cell cycle markers. Canonical cell cycle markers are highly expressed among B cells than in any other cell cycle population in SiNETs [74]. Despite the importance of B lymphocytes in the adaptive immunity response, more research is needed concerning B-cell relationships with T cells and potential therapeutic applications of B cells in GEP-NETs.

#### 2.1.9. Clinical Trials on the TIME

With the present understanding of the TME in GEP-NETs, and specifically the roles of each immune cell population promoting tumor progression, current and future studies will be aimed at targets within the TME, beyond current surgical resection and locoregional therapies. Early TME therapy research involved the implementation of interferon therapy. Interferons are intercellular signaling proteins that assist the body’s immune system in combating diseases. Among all interferons, the role of interferon alpha (IFN-alpha) has been studied the most in GEP-NET biology and as a potential treatment focus [75]. In patients, tumors treated with IFN-alpha experienced 25 months of PFS, while most experienced 3 months of stable disease. Additionally, several patients presented either partial or complete responses [76]. However, combining IFN-alpha with other therapies did not improve patient outcomes. For instance, IFN-alpha was shown to be more effective than somatostatin analogs (SSAs), among higher-grade SiNETs possessing lower somatostatin receptor expression. However, when in combination with the SSA, octreotide, the disease was stable, with no significant decrease in tumor growth [77]. Furthermore, combining drugs including Streptozotocin and Doxorubicin (Adriamycin^®^) with alpha-IFN-2a showed no progress in treatment efficacy compared to alpha-IFN-2a alone, while other drugs such as Bevacizumab (Avastin^®^) held longer treatment efficacy than alpha-IFN-2a [78]. While some positive outcomes have been observed, notable IFN-dependent toxicities are noted, including life-threatening autoimmune reactions and IFN antibody resistance. While the reasons of IFN resistance are unclear, studies show that the overexpression of the suppressor of cytokine signaling protein 1 (SOCS1) may be involved [28].

Immune checkpoint blockade (ICB) is a branch of immunotherapy with significant research and clinical interest (Table 2). These therapies often consist of a monoclonal antibody that targets the immune cell and tumor cell immunorecognition “handshake”, which is often altered by tumor cells to evade detection. An example is Pembrolizumab (Keytruda^®^), which targets the immune checkpoint inhibitor programmed cell death protein 1 (PD-1) on immune cells. A phase I clinical trial using Keytruda^®^ against PNETs led to a 12% objective response rate (ORR) [79]. This same treatment was tested among other GEP-NETs in a phase II trial but presented a much lower ORR of 3.7%, with a 6-month PFS of 39.3% [80]. While the ORR is low, this could be due to the majority of this patient cohort having metastatic disease and being already heavily pre-treated, resulting in possible reduced sensitivity and increased resistance. However, given the results from these clinical trials, Keytruda^®^ has faced questions about its efficacy in GEP-NETs. Spartalizumab, which also targets the PD-1 receptor, was studied among patients with advanced NETs, including GEP-NETs [81]. The results of this study presented a 7.4% partial response for 65 patients with GEP-NETs, followed by 55.8% stable disease. However, it must be taken into consideration that the enrolled patient cohort had experienced prior therapy treatments, including Everolimus (Afinitor^®^). Therefore, the efficacy of Spartalizumab alone is still unclear. Other trials testing combined immunotherapy Ipilimumab (Yervoy^®^) (CTLA-4 monoclonal antibody) with Nivolumab (Opdivo^®^) (PD-1 targeting antibody) in PNETs revealed an ORR of 0% and 44% for low- and high-grade tumors, respectively, while most patients experienced toxicities, including hyperthyroidism, fatigue, and nausea [82]. A second analysis of a clinical trial for the same combination was tested on patients with all PNETs, providing a 43% ORR, with a 0% ORR for low-grade tumors observed again. Additionally, 66% of the total 29 patients experience immune-related toxicities, with 34% showcasing grade 3/4 events [83]. A new phase II clinical trial combined Regorafenib, a multikinase inhibitor, with Avelumab, a PDL-1 inhibitor, among advanced GEP-NETs. This study presented a 6-month ORR of 18% with a median PFS of 5.5 months. This is an example study that demonstrates that combination immunotherapies remain promising but there is room for additional investigations [84]. However, at the moment, while the efficacy of immunotherapies has been investigated in patients with GEP-NETs, few studies have reached their endpoints, and current immunotherapeutic modalities are not clinically approved. Most GEP-NETs possess an immune “cold” microenvironment, making these tumors significantly resistant to immunotherapy platforms.

**Table 2:** Summary of current therapies targeting the TME in GEP-NETs.

Therapy Type	Patient Population	Therapy	Therapy Class and Target	Primary Endpoint
Immunotherapy	Well-differentiated or moderately differentiated PNETs GEP-NETs	Pembrozilumab (Keytruda^®^)	Monoclonal antibody for PD-1	Phase 1: 12% ORR [79] Phase 2: 3.7% ORR and 39.3% 6-month PFS [80]
	Advanced GEP-NETs	Spartalizumab	Monoclonal antibody for PD-1	Phase 2: 7.4% partial response and 55.8% stable disease [81]
	All PNETs Low/intermediate PNETs	Ipilimumab (Yervoy^®^) + Nivolumab (Opdivo^®^)	Monoclonal antibody for CTLA-4 (Ipi) and PD-1 (Nivo)	Phase 2: 43% ORR [83] Phase 2: 0% ORR [82]
	Advanced GEP-NETs	Regorafenib + Avelumab	Multikinase Inhibitor and PD-1	Phase 2: 18% ORR 5.5-month PFS [84]
Anti-angiogenic therapy	GEP-NETs	Sunitinib (Sutent^®^)	Receptor tyrosine kinase inhibitor for VEGFR, PDGFR, and RET.	Phase 2: 81.1% 1-year survival rate [85] Phase 3: 9.3% ORR [86]
		Surufatinib	Small-molecule tyrosine kinase inhibitor for VEGFR, FGFR1	Phase 1b/2: 19% ORR and 92% PFS [87]
		Axitinib (Inlyta^®^)	Small-molecule kinase inhibitor for VEGFR, c-Kit, PDGFR	Phase 2: 70% Stable Disease [88]
	GEP-NETs Lower-grade PNETs	Pazopanib (Votrient^®^)	Receptor tyrosine kinase inhibitor for VEGFR, PDGFR, c-KIT, FGFR	Phase 2: 59.5% 6-month PFS [89] Phase 2: 21.9% ORR [90]
	GEP-NETs	Cabozantinib (Cabometyx^®^)	Small-molecule tyrosine kinase inhibitor for HGFR, VEGFR2, AXL, RET, FLT3	Phase 3: 5% ORR extrapancreatic NET, 19% pancreatic NET [91]
	PNETs (VHL mutation)	Belzutifan (Welireg^®^)	Small-molecule inhibitor for HIF-2α	Phase 2: 91% ORR [92,93]
	Low-grade PNETs Advanced extra-PNETs	Bevacizumab (Avastin^®^) + Temozolomide (Temodar^®^) + Temsirolimus (Torisel^®^)	Monoclonal antibody for VEGF	Phase 2: 15% ORR, 14.3 months PFS [94] Phase 2: 41% ORR, 13.2 PFS [95] Phase 2: 2% ORR, 7.1 months PFS [96]
Anti-angiogenic + Immunotherapy		Bevacizumab (Avastin^®^) + Atezolizumab (Tecentriq^®^)	Monoclonal antibody for VEGF (Bev) and PD-L1 (Atez)	Phase 2: 20% ORR [97]

### 2.2. Vascular Cells

Vascular endothelial cells (VECs) are the primary cellular components of the blood vessels’ inner linings. VECs form tight junctions to avoid fluid leakage and, as a result, prevent tumor cell intravasation and extravasation. However, due to dysfunction during cancer progression, VECs can proliferate and migrate to promote angiogenesis and tumor metastasis [98] (Figure 1). Other VEC contributions include the secretion of pro-tumor paracrine factors and the endothelial–mesenchymal transition, which are associated with cytoskeletal remodeling to further disrupt the endothelial barrier. VECs are highly heterogeneous due to the genetic and epigenetic disruption of gene expression. Morphological, functional, and behavioral changes resulting from different gene expression profiles have been noted in VECs. Such heterogeneity is often influenced by the Notch/VEGFR signaling pathway, protein kinase D signaling pathway, or activities of the Fox01 transcriptional factor [99].

Studies on the vasculature of highly vascular GEP-NETs remain important. PNETs were shown to have enclosed vasculature tufts (EVTs), which typically have clusters of microvessels, within an insulated vasculature, containing CD31+, CD34+ endothelial cells, αSMA+ pericytes, and CD34+ stromal cells separated from tumor neuroendocrine cells. However, it is secondary modifications, such as fibrosis and calcification, that are used to indicate EVT signs in PNETs [100]. Certain hereditary genomic conditions can also affect tumor vascularization, including mouse model studies to explain the increased vascular density among PNETs with MEN1 syndrome [101]. However, these studies remain insufficient or inconclusive, leading to a need for future investigations [102]. Similarly to angiogenesis, lymphangiogenesis is another characteristic among PNETs influenced by the enzymatic activity of heparanase. This enzyme is a significant tumor factor, since its high expression correlates with advanced tumor stage, grade, and distant metastasis (Table 1) [31]. Furthermore, the presence of hemorrhagic regions (small blood-filled chambers resulting from epithelial cell detachment) is another aspect to consider during metastasis formation, as has been shown among PNETs. Though the cause is unknown, current studies suggest a possible correlation between hemorrhagic regions and E-cadherin/β-catenin overexpression in tumor cells [103].

There are several interesting observations on the signs of VEC expression through various factors among PNETs. However, more studies are required based on what has already been established about VECs from other cancer types. Given the established role of VEC in cytoskeletal remodeling, studies should analyze this further in GEP-NETs and their linkage to metastasis formation. The relationship of VECs with genetic variations found among other cancers also indicates that studies should consider this observation among GEP-NETs. Given the dependence of VEC activity on genetic profiles, the question arises about the variations in VEC activity among PNETs with different mutations. While some studies have considered MEN1 mutations and VEC activity, other major GEP-NET mutations should be explored. Similarly, the role of EVTs towards fibrosis and calcification is still unknown, though studies show their correlation with each other among PNETs. Additional analyses are needed on how cellular profiles (i.e., CD31+, CD34+) interact with EVTs. For more mechanistic analyses, pre-clinical models are essential. Factors contributing to the highly vasculature characteristic of GEP-NETs, including the enzyme heparanase, should also be validated.

A significant research effort has been placed into investigating the role of vascular endothelial growth factor (VEGF) in GEP-NETs. Certain matricellular proteins play a role in VEGF/VERGFR-2 dependent tumor angiogenesis and may represent future novel therapeutic targets. However, their function needs further validation as the only protein, CYR61, was discovered in PNETs to promote angiogenesis through VEGF expression, as studied in Rip1Tag2CYR mice [32]. Furthermore, VEGF expression in gastrointestinal NETs significantly contributes to high intratumoral vascular density as demonstrated using orthotopic xenograft models and Rip1Tag2 transgenic mouse models [104]. Similarly, VEGFC expression upregulated by c-MYC expression promotes lymphangiogenesis [33]. Given this, further analysis should be carried out in looking into the role of heparanase in VEGFC expression for lymphangiogenesis among PNETs (Table 1).

These studies show that even though VEGF may be an effective marker for angiogenesis, there are contradicting observations as to whether it has a role in the treatment of these tumors and their metastasis. One study showed VEGF is secreted by PNET cells, and the usage of a VEGF antibody, Bevacicumab, Avastin^®^, exhibited growth inhibition, thus suggesting VEGF’s role in tumor growth [105]. Another showcased PNET cells with a high expression of VEGFR-2; however, its reduction enhanced the cell proliferation of the PNET cell line [106]. In vivo studies, however, using the RIP1-Tag2 mouse model exhibited increased growth with the presence of VEGF-B, along with an increase in blood vessel diameter. Among SiNETs, it was studied that the reduced expression of VEGFR-2 was driven by the silencing of receptor neuropilin-2 (NRP-2), causing the activation of various oncogenic pathways [34]. Given these observations, further studies should not only clarify the role of VEGF on GEP-NET growth but also consider the different subtypes individually. Different VEGF subtypes have contradictory roles, and therefore, each role of those subtypes must be elucidated first. For instance, VEGFR-2 in particular is shown to be tumor-supportive when reduced, which should be validated among mouse models besides cell lines.

Similarly to VEGF, HIF-1 has been known to drive tumor formation and contribute to angiogenesis and has been the focus of research in GEP-NETs. Among GEP-NET patients, it is observed that patients with an increased tumor expression of HIF-1α have a significantly lower median OS [107]. Presently, sumoylation-enhancing protein (RSUME) is shown to stimulate HIF-1α expression and promote VEGF-A vascularization. Despite this finding, studies have only demonstrated RSUME’s function among PNET cell lines. Others have also observed a reduction in this expression among insulinomas, suggesting a different factor that could also promote HIF-1α expression or angiogenesis in this tumor type [35]. Another oncogenic transcription factor, Cut homeobox 1 (CUX1), has been shown to promote angiogenesis and is upregulated during tumor progression among insulinomas **(**Table 1). Its upregulation also strongly correlates with HIF1α upregulation, along with MMP 9 [36]. Given this factor variation, despite the same tumor type, more studies are needed to find alternative effective anti-angiogenic targets. HIF-1α expression is a factor of interest regarding VEGF-A expression. However, mechanisms of HIF-1α are not fully known, showing variation among PNETs and other GEP-NETs. Further analysis is needed on this, including an analysis of the RSUME function to effectively target HIF-1α in reducing VEGF-A vascularization. Similarly, this applies for CUX1.

GEP-NETs are highly vascular tumors, with the expression of several pro-angiogenic factors (e.g., STAT3, VEGF, and HIF-1α) shown to have increased expression among higher-grade NETs [108]. Therefore, the altered expression of these factors may lead to therapeutic targets in difficult-to-treat high-grade tumors. PDGFRα and PDGFRβ are prominently expressed in higher-grade GEP-NETs and represent novel targets for anti-angiogenic treatment [37,38] Some PNET mouse models have shown pericytes, cells located between the endothelial cells of capillaries, expressing α-SMA as a biomarker for determining the efficacy of anti-angiogenic therapy [109]. Pericyte distribution has been discovered as a crucial factor for determining the vascular characterization among PNETs due to its variation in the expression of several angiogenic regulators. Furthermore, other markers can be used to observe structural changes in intratumoral blood vessels among GEP-NETs. These markers, including vasohibin-1 (VASH-1), CD31, and endoglin, can predict tumor progression and prognosis [110]. Additionally, higher circulating levels of angiogenic markers, such as placental growth factor, angiopoietin 2, interleukin 8, and PROK2, are observed in GEP-NETs [89,90]. Although VEGF is the most studied angiogenic factor in GEP-NETs, there still arise controversies about its use as a marker. VEGF has been shown to correlate with increased angiogenesis, decreased PFS, and increased locoregional spread in some studies [111,112]. In other studies, however, while angiogenic markers, including endoglin, positively correlate with tumor size, the presence of metastases, and a more advanced tumor stage, VEGF does not [113]. Regardless, there is insufficient data on angiogenic markers to determine disease stage and treatment outcomes [114]. Though studies present several angiogenic markers for GEP-NETs, there still needs to be data demonstrating its correlation to VEGF expression and whether the use of such markers can help determine increased vasculature and tumor growth through VEGF presence.

Alongside the contradictory role of VEGF, finding multiple effective angiogenic targets for GEP-NET therapy is crucial, as some angiogenic factors predominantly enhance metastasis and micro-vessel density, but not primary tumor growth. For example, angiopoietin-2 (Ang-2), a ligand of the endothelial tyrosine kinase Tie-2, increased microvessel density and lymphatic metastasis, but did not affect primary tumor growth. This raises the question of whether tumor angiogenesis has a direct relationship with cell proliferation and if combination therapy would be a more effective strategy for decreasing tumor angiogenesis and tumor growth [115].

#### Clinical Trials Targeting VECs

Increased VEGF expression and serum levels among low-grade NETs often correspond to angiogenesis and decreased PFS. Blocking the production of VEGF has been extensively tested in clinical trials for GEP-NETs. Previous research has demonstrated that inhibiting VEGF, either using a single anti-VEGF antibody, such as Avastin^®^, or in combining with other therapies, may be an effective therapeutic route [116] (Table 2). Avastin^®^ selectively binds to VEGF, preventing it from binding to its associated receptors. Targeting VEGF activity results in the growth inhibition of tumor-associated blood vessels. Avastin^®^ has often been studied in combination with other agents, including Temozolomide (Temodar^®^) (15% ORR and 14.3 months PFS) and Temsirolimus (Torisel^®^) (41% ORR and 13.2 months PFS) [94,95]. However, more recent studies with Torisel^®^ in extra-pancreatic NETs presented the increased toxicity and minimal efficacy of this combination, as shown by 2% ORR and 7.1 months of median PFS [96]. At the moment, the reason for this is unclear and requires additional patient cohorts for further study. Combining Avastin^®^ with immunotherapy presents an intriguing treatment opportunity, as demonstrated in a clinical trial utilizing Avastin^®^ with Atezolizumab (Tecentriq^®^). Among PNET patients, this study presented an ORR of 20% [97]. While some of these studies did show a decrease in tumor blood flow and improved PFS, additional studies with a higher number of patients are needed to confirm treatment efficacy [117]. Despite identifying several therapeutic targets to date, personalized anti-angiogenic applications may be necessary, and novel combination therapies must be further studied for improved efficacy [118].

Given that many NETs are highly vascular, treatments should aim to target pathways activated by vascular growth factors. While these therapies may not always directly target VECs, they can often disrupt the signaling between VECs and tumor cells. Sunitinib (Sutent^®^), a pan-tyrosine kinase inhibitor, is a widely studied drug in GEP-NETs, having activity against VEGFR, PDGFR, and RET. In a phase II clinical study, patients receiving Sutent^®^ had an 81.1% one-year survival rate [85]. Phase III trial data demonstrated an ORR of 9.3% in the Sutent^®^-treated group compared to 0% in the placebo group [86]. Given these results, Sutent^®^ is now FDA-approved for the treatment of advanced PNETs. Similarly, Surufatinib, an inhibitor targeting VEGF, fibroblast growth factor receptor (FGFR) 1, and CSF1, was shown to have a 19% ORR and PFS of 92% in a clinical trial performed in China [87]. In addition, Surufatinib presents only common grade 3 treatment-related adverse events, unlike other VEGF inhibitors. The VEGF inhibitor, Axitinib (Inlyta^®^), presented a 70% stable disease response but demonstrated a high number of grade 3/4 adverse effects [88]. Cabozantinib (Cabometyx^®^), a multitargeted agent that inhibits platelet-derived growth factor (PDGF), VEGFR2, c-Kit, and Flt3, was recently tested in a phase III trial comparing efficacy to a placebo [91]. The ORR for non-pancreatic tumors was at 5%, while pancreatic tumors demonstrated a rate of 19% compared to the placebo, whose ORR was 0% for both groups. A similar therapy, Pazopanib (Votrient^®^), demonstrated efficacy in 59.5% of 44 patients, with a PFS rate of 6 months, among low-grade PNET patients [89]. While 21.9% of 52 patients experienced an ORR, there were significant toxic effects, including grade 4 hypertriglyceridaemia, grade 4 thrombosis and a few grade 3 events. Further assessment is needed on the therapeutic effect of Votrient^®^ among GEP-NETs [90]. HIF transcription factor inhibition is another therapeutic target possibility, as it is responsible for hypervascularization in VHL mutant patients. In a phase II trial, an HIF-2α inhibitor, Belzutifan (Welireg^®^), was tested among VHL-mutant PNET patients, resulting in a 91% ORR [92,93]. Based on these results, Welireg^®^ is FDA-approved for the treatment of VHL-mutant PNETs.

Although numerous clinical and basic science studies have described the angiogenic process in GEP-NETs, most studies have focused solely on PNETs. Future GEP-NET anti-angiogenic studies will benefit from SiNET and GasNET involvement.

### 2.3. Fibroblasts

Fibroblasts are a subpopulation of stromal cells that maintain and form connective tissue to support tissue types throughout the body. Fibroblasts also hold prominent roles in tissue repair, wound healing, and the coordination of other cell activities [119]. In cancer, fibroblasts may undergo a transformation into cancer-associated fibroblasts (CAFs), which promote tumor progression through several functions. Through the secretion of growth factors, pro-angiogenic molecules, chemokines, and cytokines, CAFs influence tumor cell proliferation, angiogenesis, treatment resistance, and immunosuppression. CAFs also play a prominent role in ECM remodeling through regulating the expression of tissue remodeling enzymes, including lysyl oxidases, matrix metalloproteinases (MMPs), and transglutaminases (Figure 1). As a result, ECM remodeling leads to matrix stiffness, which contributes to treatment resistance or matrix degradation, ultimately leading to cancer cell invasion [120].

CAFs have been shown to have multiple potential phenotypes, based on CAF characteristics and their role in tumor progression. Several markers are currently used to detect the presence of CAFs. A prominent marker among PNETs is alpha-smooth muscle actin (α-SMA), which is expressed in all tumor grades. For instance, grade 3 tumors possess a significant upregulation of matrix metalloproteinases and collagen expression genes influenced by CAFs, which promote tumorigenic activities, specifically ECM remodeling. Studies show that CAF tumor influence is directly correlated with an increased expression of α-SMA [121]. Recent studies have shown three phenotypes among NF-PNETs through single-cell technologies and immunohistochemistry (IHC): myofibroblastic CAFs (myCAFs) are responsible for tissue repair and wound healing, inflammatory CAFs (iCAFs) are involved in tumor-associated inflammation, and antigen-presenting CAFs (apCAFs) regulate tumor immunity [122]. Spatial transcriptomics studies have provided a comparative analysis of CAF subpopulations between primary and metastatic tumors, with PNETs presenting increased CAF population diversity in primary tumors than in the liver metastases [123]. Further understanding the heterogeneity and subtype distributions of CAFs among NETs will assist in possibly identifying specific secreted markers contributing to invasion and metastasis formation.

In addition to their different phenotypes, differing CAF tissue sites of origin demonstrate varying responses to tumor spread. When cultured with the SiNET STC-1 cell line, fibroblasts originating from the dermal and colon regions exhibited limited spreading potential, while those from the jejunum, liver, and lung regions spread to form trabecular layers [124]. This differential spread may indicate diverse CAF populations with unique effects on tumor spread.

CAFs’ influence on growth and therapy resistance towards NETs has become a topic of research interest. Using various co-culture techniques, with fibroblast conditioned media, recent studies have begun to elucidate a relationship between CAFs and increased PNET cell line growth and therapeutic resistance. It has been demonstrated that increased proliferation among PNET BON-1 and QGP-1 cell lines cultured in CAF-conditioned media may be prevented using the mTOR inhibitor Afinitor^®^. The study showcases a targetable pathway that may be upregulated in CAF–tumor co-culture and plans to implement a strategy to overcome any treatment resistance against everolimus [39]. Contrary to this, others have discussed CAF contribution to increased growth among BON-1 and NT-3 cell lines and resistance to Afinitor^®^ through the activation of the STAT3 pathway [40]. Silencing STAT3 would prevent this CAF-influenced treatment resistance. While these studies show promise, further efforts are needed to elucidate the connection between CAF- altered pathways and therapy responses.

CAF-secreted growth factors play a prominent role in GEP-NET biology. TGF-β, for instance, is a prominent growth factor secreted by fibroblasts and is widely studied in GEP-NETs [125]. While tumor cells expressed three isoforms of TGF-β (β-1, β-2, and β-3), strong signals of TGF-β2 were observed among stromal cells, in addition to the TGF-β binding protein in GEP-NETs [41]. Platelet-derived growth factor (PDGF) and basic fibroblast growth factor (bFGF) have also shown significant differences in expression, where one isoform is strongly expressed on tumor cells, compared to stroma, and vice versa for the other [42,43,44]. Recent PNET research has demonstrated that PDGF is involved in CAF recruitment, ECM deposition, and the promotion of TGF-β signaling [126]. While the CAF secretion of growth factors assists in the growth of tumor cells, studies show that growth factor secretion by CAFs aids their own growth promotion as well. Growth factors such as thrombin, bombesin, bradykinin, and vasopressin activate phospholipase C to assist with the stimulation of DNA synthesis in fibroblasts [45]. Similarly, NET amines, biogenic amines that play roles as neurotransmitters and hormones, also have an influence, demonstrated by 5-HTs, serotonin receptor binding proteins, and function towards fibroblast proliferation in SiNETs [46]. Finally, stromal cell-derived factor-1(SDF-1) is secreted by CAFs and is influenced by interleukin-1 (IL-1) leading to an elevated expression of anterior gradient-2, leading to PNET growth and metastasis (Table 1) [47].

Though it is understood that CAFs have a direct involvement in increased growth among PNETs, there needs to be further research on CAFs’ involvement in GasNETs. In addition, a further analysis of the interaction of growth factors between CAFs and tumor cells is needed. While basic scientific studies can demonstrate this with a few targetable factors, there needs to be translational approaches to determine therapeutic efficacy among these pro-tumor markers. This includes considering factors that may affect tumor growth by CAFs, potential effects in CAF–tumor interactions due to differences in tumor grade and location, and altered isoforms of growth factors secreted by CAFs.

#### Therapeutic Targeting of Cancer-Associated Fibroblasts

Therapies involving the direct targeting of CAFs are limited. While CAFs present potential targets for therapeutic intervention, these targets are often present on healthy and tumor cells, making it difficult to determine potential efficacy [127] (Table 2). Instead, it may be prudent to reduce the effect of growth factors on the tumor cell by targeting the tumor cell receptors. As stated above, therapies targeting the PDGF, TGF-β, and FGF pathways in cancer cells have undergone extensive clinical trials, with several approved in other cancers, and even some have been tested in GEP-NETs, such as Sutent^®^, Votrient^®^, and Cabometyx^®^. While their efficacy has not been confirmed in GEP-NET CAFs, there will likely be anti-tumor activity in these tumors after therapeutic targeting.

### 2.4. Extracellular Matrix (ECM)

The extracellular matrix (ECM) is composed of several macromolecules spatially organized into a supportive network. The composition and structure of the ECM varies based on tissue type and function, ranging from stiff, as seen in the skeleton, to softer formations, such as found in the brain [128]. The components of the ECM can include collagens, proteoglycans, laminin proteins, fibronectin, elastin, and several ECM molecule receptors, such as integrins and syndecans. This matrix provides a stable structure for cell attachment and formation, providing mechanical properties and cues for many cellular interactions. During tumor progression, the ECM often transforms into a more favorable TME. This process can occur through the regulation of matrix remodeling enzymes secreted by the TME cellular components. The ECM is constantly being remodeled and often increases in tensile strength and matrix stiffness according to tumor grade.

Several methods can be utilized to analyze ECM composition, and these analyses can give insight into TME remodeling compared to normal tissue. To assess ECM proteins, quantitative profiling was performed on PNETs compared to a matched normal pancreas. Of the 120 proteins detected, 35 were found to be significantly differentiated, including EGF-containing fibulin-like extracellular matrix protein 1 (EFEMP1), fibrillin 1, and periostin, which were high in abundance in the tumor, while decorin, deleted in malignant brain tumor 1 (Dmbt1), hemicentin, and Vwa5 were lower in comparison to normal pancreas tissue [129]. Compared to PNETs, using differential gene expression analysis, SiNETs express a distinctive gene expression profile, with a significant upregulation of extracellular matrix protein 1 (ECM 1), a protein that aids in ECM remodeling [130]. These studies show that quantitative profiling can be utilized for determining ECM markers and that variations in expression between GEP-NETs may be effective for tumor-specific biomarkers.

Several ECM remodeling enzymes are found in both healthy and normal tissues. One of the most prominent groups is matrix metalloproteinases (MMPs), consisting of 23 members. In cancer, MMPs can degrade the ECM, promoting invasion and metastasis, or contribute to matrix stiffness, depending on the levels of their tissue inhibitor of MMP (TIMP), which consists of several subtypes controlling various MMPs. Given the expression of MMPs and their TME-specific role towards the ECM, they serve as biomarkers in understanding the tumor phenotype of GEP-NETs. In PNETs, MMP-2 expression serves as a prognostic marker for aggressiveness, alongside MMP-9 [131]. This is due to the expression of thrombospondin (THBS2), which inhibits the production of MMP-9 by suppressing the transcriptional activity of CUT-like homeobox (CUX1). It was also observed that the co-expression of these markers is not a significant indicator of an invasive tumor phenotype [132]. Another investigation analyzed the relationship between TIMP1 and MMP2 and showed high expression among high-grade GEP-NETs and metastatic tumors. This indicates that TIMP1 could also be a marker for metastasis but may not have a direct correlation to MMP-2 inhibition. Therefore, more studies are needed to identify specific MMPs that assist in matrix stiffness among GEP-NETs and assist in ECM degradation under the influence of TIMPs [133]. In a further analysis of MMP-2 expression among PNETs, others have studied the role of extracellular matrix metalloproteinase inducer, EMMPRIN (CD147). It was found that EMMPRIN repressed MMP-2 expression but increased MMP1 expression. This result demonstrates that the regulation of MMP inhibitors and activators varies between GEP-NETs, and differential expressions between MMP types involved are necessary to achieve a specific matrix characteristic [134].

Another important ECM enzyme is heparanase, which regulates the levels of heparan sulfate proteoglycans of the ECM. Though heparanase is not widely evaluated in all GEP-NETs, studies among PNETs demonstrated a direct correlation between high heparanase expression and advanced tumor grade and distant metastasis [31]. Finally, the von Hippel–Lindau (VHL) tumor suppressor gene, a gene found mutated in a subset of GEP-NET patients, has been found to upregulate fibronectin deposition (upregulated in the presence of VHL unaffected by hypoxia) [135].

In addition to understanding the role of these enzymes in matrix structure, studies have also investigated the changes in ECM components using 2D and 3D TME models. Work performed among the SiNET line, STC-1, shows a deposition of collagen IV and laminin 1, providing a different ECM composition to other NETs. The varying levels of proteins found in the tumor ECM can also be influenced by the fibroblast subsets residing in the tumor [124]. Given MMP’s role in metastasis, more studies are needed to compare MMP expression among primary vs. metastatic tumors. The present literature showcases a solid foundation regarding the relationship between MMPs and TIMP1 and the presence of MMPs among GEP-NETs. However, more studies are needed showing the targeting and inhibition of this expression and its effect on other cell populations, including CAFs, which are well known to interact with these factors for ECM remodeling, or immune cells that promote immunosuppression due to matrix stiffness. A few observations were made regarding the variation in ECM composition between GEP-NET types. However, these variations are still unknown, given that most studies evaluate the characteristics of PNETs. Therefore, studies should also focus on considering matrix evaluation among all NET subtypes for a better comparison.

## 3. Conclusions

Our understanding of the TME in GEP-NETs has significantly grown in recent years. Advances in TME research have improved our perspective on tumor biology, leading to potential new treatment options in the future. Additionally, work on non-cellular components, including the ECM and secreted factors, has led to a better understanding of patient tumor characteristics and outcomes. However, further research on the TME of GEP-NETs is needed due to several shortcomings.

Due to poor historical understanding and low incidence rates for GEP-NETs, there is a lack of suitable models for studying the TME. Studies among cells grown in 2D culture are insufficient for fully understanding the characteristics and behavior of a cell population in the TME as it alters their behavior, not to mention many of the cell lines represent more aggressive neuroendocrine carcinomas, a cancer with a significantly different tumor biology [10,11,136]. Furthermore, there are speculations regarding changes in mutational profiles over time for cell lines, further questioning their reliability. Some suitable animal models exist; however, most are developed from either heavily mutated “carcinoma”-like tumors, germline knockouts that develop tumors spontaneously and thus are difficult to standardize, or are immunocompromised, which hinders the study of the immune system. Though animal models are highly preferred for the whole-system analysis of therapies, generating these models for NETs has shown tremendous challenges. Alternatively, 3D in vitro models may provide a spatially accurate representation of the TME and cell population interactions. They are less time-consuming and cost-efficient to generate and allow for more direct observations of smaller numbers of cell populations and fine-tuned ECM structure. Three-dimensional models have been shown to be more readily generated from biospecimens, including for immune co-cultures in studying therapy response in other rare cancers [137,138,139]. Shortcomings of 3D cultures include the standardization of culture conditions across research groups and ensuring biologically relevant cell behavior. Regardless, this model type is promising in comparison to 2D culture and even animal models. Studies are now aiming to prioritize these models to incorporate tumor heterogeneity, given its importance.

Following the development of therapies based on TME-based research, several issues present during the development of clinical trials, which may hinder our understanding of therapy response in GEP-NET patients. The first limitation is low patient accrual due to low incidence rates and complex tumor biology [140]. This leads to multiple issues including low statistical power, poor recruitment into treatment arms, and difficulties in creating balanced treatment arms. Because of the limited biomarkers available, patient selection into treatment arms has been previously based on tumor progression and less on potential prognostic biomarkers, in particular for non-functional tumors [141]. Furthermore, those at advanced stages have often received multiple lines of therapy previously, resulting in issues resolving therapy efficacy between arms, the presentation of low response rates to therapies, and transference between treatment arms during clinical trials. Additionally, patient toxicity has remained a major issue across studies, especially for immunotherapies in clinical trials. Due to the indolent nature of many GEP-NETs, patients may experience severe side effects before seeing an effective reduction in tumor size, leading to further risk to the patient cohort’s health in studies. While newer studies have incorporated more precise methods of clinical trial design, further work is needed to rectify these issues to create more accurate study designs [142].

Thus, while significant progress has been made towards elucidating novel TME therapeutic targets in GEP-NETs, further work is needed to improve future clinical trials to fully utilize the targeting of the TME for improved patient outcomes.
